# HDL-cholesterol concentration in pregnant Chinese Han women of late second trimester associated with genetic variants in *CETP, ABCA1, APOC3,* and *GALNT2*

**DOI:** 10.18632/oncotarget.18128

**Published:** 2017-05-24

**Authors:** Mingxuan Cui, Wei Li, Liangkun Ma, Fan Ping, Juntao Liu, Xueyan Wu, Jiangfeng Mao, Xi Wang, Min Nie

**Affiliations:** ^1^ Department of Endocrinology, Peking Union Medical College Hospital, Peking Union Medical College, Chinese Academy of Medical Sciences, Key Laboratory of Endocrine, National Health and Family Planning Commission, Beijing, China; ^2^ Department of Obstetrics & Gynecology, Peking Union Medical College Hospital, Peking Union Medical College, Chinese Academy of Medical Sciences, Beijing, China

**Keywords:** high-density lipoprotein-cholesterol, pregnancy, polymorphisms, miRNA-binding site polymorphisms, HDL-C-related genes

## Abstract

**Objective:**

To investigate whether HDL-C level in pregnant Chinese Han women of late second trimester correlated with loci in high-density lipoprotein-cholesterol (HDL-C)-related genes found in genome-wide association studies (GWAS).

**Methods:**

Seven single-nucleotide polymorphisms (rs3764261 in *CETP*, rs1532085 in *LIPC*, rs7241918 in *LIPG*, rs1883025 in *ABCA1*, rs4225 in *APOC3*, rs1059611 in *LPL*, and rs16851339 in *GALNT2*) were genotyped using the Sequenom MassArray system for 1,884 pregnant women.

**Results:**

The following polymorphisms were statistically associated with HDL-C level after adjusting for age, gestational week, pre-pregnancy BMI and state of GDM or HOMAIR: (i) rs3764261 (b = -0.055 mmol/L, 95% CI -0.101 to -0.008, *p* = 0.021), (ii) rs1883025 (b = -0.054 mmol/L, 95% CI -0.097 to -0.012, *p* = 0.013), (iii) rs4225 (b = -0.071 mmol/L, 95% CI -0.116 to -0.027, *p* = 1.79E-3) and (iv) rs16851339 (b = -0.064 mmol/L, 95% CI -0.120 to -0.008, *p* = 0.025). The more risk alleles the pregnant women have, the lower the plasma HDL-C levels of the subjects are.

**Conclusions:**

Several risk alleles found to be related to HDL-C in GWAS are also associated with HDL-C levels in pregnant Chinese Han women and these risk loci contribute additively to low HDL-C levels.

## INTRODUCTION

During pregnancy, high concentrations of total cholesterol, triglycerides (TG), low-density lipoprotein-cholesterol (LDL-C), high-density lipoprotein-cholesterol (HDL-C) levels are common [1]. Studies have shown that serum lipid levels begin rising from the 9^th^ to 13^th^ week of gestation, reach the peak at the 31^st^ to 36^th^ week, maintain at the plateau until childbirth, and finally decline to normal levels 4 to 6 weeks postpartum [2]. Lipid profile disorders are recognized to be involved in the pathophysiology of cardiovascular disease and diabetes [3–6]. HDL-C is a well-known protective lipoprotein. Low plasma HDL-C is an independent risk factor for multiple diseases during pregnancy, including coronary heart disease (CHD), diabetes, and preeclampsia [3, 7]. Decrease in maternal HDL-C levels could also affect fetal programming and susceptibility to chronic diseases in later life [4, 5].

Environmental and genetic factors are important in determining HDL-C levels and the estimated heritability of blood lipid levels is 40–70% [6, 8]. Therefore, the hereditary basis of HDL-C levels was investigated intensively using genome-wide association studies (GWAS) and mitochondrial GWA analysis [9]. To date, 71 single-nucleotide polymorphisms (SNPs) in 71 genes have been found to be associated with HDL-C levels [10] and a few have been verified in the Chinese population [11, 12]. Due to the differences in genome-wide linkage disequilibrium patterns between different ethnic groups and the changes in lipids that occur during pregnancy, it is unclear whether the loci identified in European GWAS also exert similar effects on lipid levels among the pregnant Chinese Han population.

Currently, an increasing number of studies have revealed that microRNAs (miRNAs) play critical roles in various physiological processes. MiRNAs are endogenous, small noncoding RNA molecules about 20 nucleotides in length, that function by binding to the 3′-untranslated region (3′UTR) of target gene mRNAs to regulate gene expression. Recent evidence shows that different miRNAs regulate the expression of target gene through binding to different genotype of the specific SNPs in 3′UTR of the target gene [13]. These kinds of SNPs in miRNA-binding sites of 3′UTR are known to contribute greatly to disease susceptibility, such as angiocardiopathy and diabetes [14, 15].

In this study, we aimed to investigate whether the loci in HDL-C-related genes identified by GWAS and miR-binding SNPs are correlated with HDL-C level in pregnant Chinese Han women.

## RESULTS

The clinical characteristics of the pregnant subjects are shown in Table 1. The median of age, gestational week, fasting plasma glucose, pre-pragnancy BMI, HOMAIR, fetal birth weight, total cholesterol, TG, HDL-C, and LDL-C was 31 (yrs), 27.71 (w), 4.60 (mmol/L), 21.09 (kg/m^2^), 1.44, 3.32 (kg), 6.07 (mmol/L), 2.36 (mmol/L), 2.02 (mmol/L) and 3.31 (mmol/L), respectively.

**Table 1:** Clinical characteristics of all subjects

	Median	QR	
		P25	P75
Age (years)	31	29	34
Gestational week (weeks)	27.71	26.43	28.86
Fasting plasma glucose (mmol/L)	4.60	4.33	4.90
pre-pregnancy BMI (kg/m^2^)	21.09	19.36	23.19
HOMAIR	1.44	0.96	2.20
Fetal birth weight (kg)	3.32	3.02	3.63
Total cholesterol (mmol/L)	6.07	5.36	6.77
TG (mmol/L)	2.36	1.88	2.98
HDL-C (mmol/L)	2.02	1.75	2.30
LDL-C (mmol/L)	3.31	2.76	3.92

Data is reported as median (interquartile range) for the quantitative variables with a non-normal distribution.

The allelic and genotype frequencies of the seven SNPs were in Hardy-Weinberg equilibrium in all subjects (*P* > 0.05). The three miR-binding SNPs (rs4225, rs1059611, and rs16851339) were predicted to interact with hsa-miR-486-3p, hsa-miR-136, and hsa-miR-1260, respectively (Table 2).

**Table 2:** SNPs in the 3′UTR of HDL-related genes and predicted miRNA binding partner

SNP (gene)	Minor/major allele	Chromosome location	MAF in 1000 genome (CHB)	Predicted binding miRNA	Algorithm
rs4225 (*APOC3*)	T/G	11:116832955	0.189	hsa-miR-486-3p	SNP Function Prediction&TargetScan
rs1059611 (*LPL*)	C/T	8:19967052	0.097	hsa-miR-136	SNP Function Prediction&microRNA.org
rs16851339 (*GALNT2*)	A/T	1:230281086	0.097	hsa-miR-1260	SNP Function Prediction&miRDB

MAF: minor allele frequency; CHB: Han Chinese in Beijing.

The frequency and the HDL level of each genotype under the additive model are presented in Table 3. After nonparametric data analysis, we discovered that HDL level was significantly associated with rs3764261 (*p*= 6.70E-6), rs1532085 (*p*=3.18E-3), rs7241918 (*p*= 0.0482), rs1883025 (*p*= 5.06E-3), rs4225 (*p*= 9.36E-7) and rs16851339 (*p*= 0.0149). No association with HDL level was observed with respect to rs1059611.

**Table 3:** Comparison of HDL-C level among pregnant Chinese Han women with different genotype of specific SNP

SNP (gene)	Genotype	Subject number (%)^a^	HDL level^b^	Additive model *P* value^c^
rs3764261 (*CETP*)	AA	58(3)	2.29(1.99, 2.56)	**6.70E-6**
	AC	506(28)	2.03(1.76, 2.33)	
	CC	1246(69)	1.99(1.72, 2.27)	
rs1532085 (*LIPC*)	AA	410(23)	2.06(1.79, 2.33)	**3.18E-3**
	AG	897(50)	2.02(1.75, 2.31)	
	GG	498(27)	1.95(1.69, 2.25)	
rs7241918 (*LIPG*)	GG	22(1)	1.84(1.61, 2.15)	**0.0482**
	GT	357(20)	1.98(1.70, 2.29)	
	TT	1436(79)	2.02(1.75, 2.30)	
rs1883025 (*ABCA1*)	TT	81(5)	1.93(1.71, 2.29)	**5.06E-3**
	TC	605(33)	1.95(1.72, 2.24)	
	CC	1129(62)	2.05(1.76, 2.33)	
rs4225 (*APOC3*)	TT	69(4)	1.79(1.61, 2.16)	**9.36E-7**
	TG	559(31)	1.94(1.68, 2.24)	
	GG	1201(65)	2.04(1.78, 2.33)	
rs1059611 (*LPL*)	CC	15(1)	2.01(1.70, 2.12)	0.6628
	CT	277(15)	1.98(1.76, 2.25)	
	TT	1519(84)	2.01(1.74, 2.30)	
rs16851339 (*GALNT2*)	AA	29(2)	2.17(2.02, 2.36)	**0.0149**
	AT	347(19)	2.06(1.73, 2.34)	
	TT	1458(79)	1.99(1.74, 2.29)	

^a^The total number is different from the whole sample size 1884 because the genotyping success rates were 96.1%, 95.8%, 96.3%, 96.3%, 97.1%, 96.1% and 97.3% for rs3764261, rs1532085, rs7241918, rs1883025, rs4225, rs1059611 and rs16851339, respectively, not 100%.

^b^Data are reported as median (interquartile range) HDL level for non-normally distributed data.

^c^*P* value returned from nonparametric tests.

**Figure 1:**
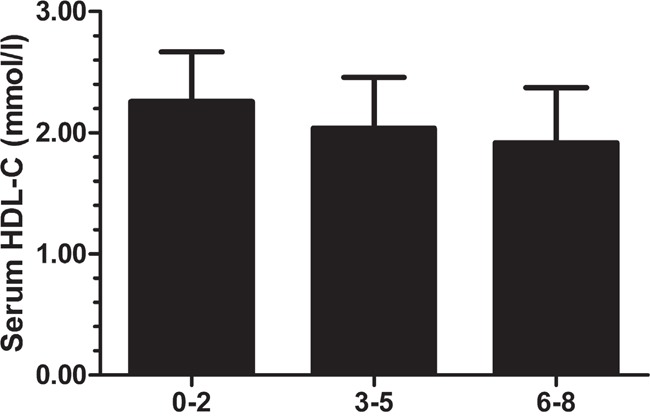
Analysis of the combined effect of multiple alleles associated with low HDL-C For each participant with complete genotype information for the 7 SNPs, the number of alleles associated with low HDL-C were counted (C of rs3764261, T of rs1883025, T of rs4225, and T of rs16851339). Subjects with 0-2, 3-5, or 6-8 alleles were grouped.

Table 4 shows the associations between HDL-C levels and the seven SNPs in a multiple linear regression model after adjusting for age, gestational week, pre-pregnancy BMI and state of GDM or HOMAIR, respectively, for the significant correlation between “state of GDM” and “HOMAIR”. The four of seven SNPs were found to be significantly associated with HDL-C levels after adjustment in both models, including rs3764261 (b = -0.055 mmol/L, 95% CI -0.101 to -0.008, *p* = 0.021), rs1883025 (b = -0.054 mmol/L, 95% CI -0.097 to -0.012, *p* = 0.013), rs4225 (b = -0.071 mmol/L, 95% CI -0.116 to -0.027, *p* = 1.79E-3) and rs16851339 (b = -0.064 mmol/L, 95% CI -0.120 to -0.008, *p* = 0.025). Subsequently, the compound effects of multiple risk alleles of susceptible loci on HDL-C were investigated. Here we only selected SNPs significantly associated with HDL-C (rs3764261, rs1883025, rs4225 and rs16851339) and calculated the compound effects by summing the number of risk alleles for each participant (0 to 8 alleles). Individuals were classified into one of three different groups according to the number of risk alleles in their genome (group1: 0-2 alleles, group2: 3-5 alleles, and group3: 6-8 alleles). We found that an increased number of risk alleles was associated with lower plasma HDL-C levels (Figure 1). Subjects in group3 had significantly lower HDL-C levels than subjects in group2 and group1, and group2 had lower HDL-C levels than group1. (median HDL-C (mmol/L) [95% CI]: group1 = 2.26 [2.16-2.35]; group2 = 2.04 [2.02-2.06]; and group3 = 1.92 [1.86-1.98]).

**Table 4:** Associations between HDL-C levels and SNPs in pregnant Chinese Han women

SNP (gene)	Effect allele/other allele	Regression analysis			
		Effect size (95%CI)	P value^a^	Effect size (95%CI)	P value^b^
rs3764261 (*CETP*)	C/A	−0.055n(−0.101,−0.008)	**0.021**	−0.056(−0.104,−0.008)	**0.023**
rs1532085 (*LIPC*)	G/A	−0.017(−0.052,0.018)	0.329	−0.017(−0.053,0.020)	0.368
rs7241918 (*LIPG*)	G/T	−0.052(−0.107,0.004)	0.068	−0.043(−0.100,0.015)	0.150
rs1883025 (*ABCA1*)	T/C	−0.054(−0.097,−0.012)	**0.013**	−0.052(−0.096,−0.007)	**0.023**
rs4225 (*APOC3*)	T/G	−0.071(−0.116,−0.027)	**1.79E-3**	−0.080(−0.126,−0.033)	**8.86E-4**
rs1059611 (*LPL*)	C/T	−0.003(−0.066,0.060)	0.917	−0.027(−0.093,0.039)	0.419
rs16851339 (*GALNT2*)	T/A	−0.064(−0.120,−0.008)	**0.025**	−0.062(−0.120,−0.004)	**0.036**

^a^ *P* values are adjusted for age, gestational week, pre-pregnancy BMI and state of GDM

^b^ *P* values are adjusted for age, gestational week, pre-pregnancy BMI and HOMAIR.

*P* values < 0.05 are shown in bold.

## DISCUSSION

Expression of several lipid related genes has been shown to be inhibited by estrogen in adipocytes such as *LPL* [16]. During pregnancy, the levels of hormones especially estrogen and progesterone change significantly. Therefore, it is necessary to explore the contribution of the HDL-C related genes identified in non-pregnancy population to the variation in serum HDL-C level during pregnancy. In this study, the association between HDL-C-related SNPs from GWAS as well as miRNA-binding sites and the risk of low HDL-C in pregnant Chinese Han women was identified for the first time. Our study shows that *CETP* rs3764261, *ABCA1* rs1883025, *APOC3* rs4225 and *GALNT2* rs16851339 are associated with HDL-C level, while the other three SNPs, including *LIPC* rs1532085, *LIPG* rs7241918 and *LPL* rs1059611, are not. And we also find that the more risk alleles a pregnant woman carries, the lower her HDL-C levels will be.

### *ABCA1* rs1883025

The *ABCA1* gene (9q31.1) encoding the ATP-binding cassette transporter A1, plays a vital role in the pathogenesis of atherosclerosis and visual loss. Over-expression of ABCA1 increases cellular lipid and cholesterol efflux and levels of plasma HDL [17]. Rs1883025 was originally identified as genetic determinants of HDL-C level by GWAS in 2010 (effect size = - 0.0243 mmol/L, *p* = 1.75 × 10^−33^) [8] and our results replicate that the A allele of rs1883025 renders Chinese pregnant women susceptible to lower HDL-C levels.

In a study of meta-analysis which aimed to characterize GWAS-identified variants in diverse population-based studies, researchers were unable to replicate the finding that *ABCA1* rs1883025 is associated with lipid levels in six different racial/ethnic groups (European American, African American, American Indian, Mexican American/Hispanic, Japanese/East Asian, and Pacific Islander/Native Hawaiian adults). The association did not have sufficient power, which is duing to mainly the smaller sample sizes of non-European American population, to detect the effect size reported in GWAS (68% power; n= 3,865) [18]. The relationship between rs1883025 and HDL-C level during pregnancy and the underlying pathogenic mechanism need to be further studied.

### *CETP* rs3764261

The role of CETP in metabolism of HDL-C is well studied. CETP facilitates the net transfer of cholesteryl ester from HDL-C to other lipoproteins and transports cholesterol from peripheral tissues to the liver and regulates the concentration of HDL-C [19]. In this study, we discovered that the G allele of rs3764261 was associated with lower HDL-C levels in pregnant Chinese Han women (b = −0.055 mmol/L), which is in accordance with the results of GWAS (b=-0.0875 mmol/L) [8] and other studies in the Indian [20], Japanese [21], and Latvian [22] populations. Supporting our findings, a previous study of the Chinese Xinjiang population found that carriers of the T allele of rs3764261 had significantly lower risk of dyslipidemia compared with carriers of the major G allele [23].

### *LIPC* rs1532085 and *LIPG* rs7241918

*LIPC* plays a key role in the lipid metabolism pathway. A study identified the effects of a gene-gene interaction between *LIPC* rs1532085 and other genome-wide SNPs on HDL-C levels thus finding that rs1532085 is an expression QTL (eQTL) of *LIPC*, a gene that is a hub in the gene-gene interaction network that regulates HDL-C levels [24]. However, in our study, we observed no association between rs1532085 and HDL-C after adjusting for age, gestational week, pre-pregnancy BMI, state of GDM and HOMAIR, which is not in accordance with the results of GWAS (effect size = -0.0375 mmol/L) [8]. A relatively lower effect (b = -0.017 mmol/L, 95% CI -0.053 to 0.020, *p*= 0.368) might result in this inconsistent conclusion.

Similar to *LIPC* rs1532085, we did not find the correlation between *LIPG* rs7241918 and HDL-C as reported previously either (effect size = -0.0339 mmol/L for G allele) [8]. This discrepancy might be attributed to racial differences and the altered physiological conditions during pregnancy [25].

Because little is known about these loci, further exploration is needed.

### *APOC3* rs4225, *GALNT2* rs16851339, and *LPL* rs1059611

Rs4225 locating in the 3′UTR of *APOC3* is found associated with HDL-C in pregnant Chinese Han women (b = -0.071 mmol/l for allele T) and hsa-miR-486-3p was predicted to be its binding miRNA. There are no studies examining the relationship between *APOC3* rs4225 and HDL-C level in any race yet, however, one study [26] recently found that the T allele of rs4225 suppressed *APOC3* translation by facilitating its binding with miR-4271, which impacts the key function of rs4225 on the plasma TG regulation. It is well known that the expression of specific target gene is regulated by numbers of microRNAs, therefore, there may be multiple kinds of miRNAs binding with the SNP rs4225 to influence the plasma lipid level.

We also found that the T allele of *GALNT2* rs16851339 contributes to lower HDL-C levels (b = -0.064 mmol/L). Genome-wide SNP association studies have identified the GalNAc-transferase polypeptide gene, *GALNT2*, as a candidate gene for low HDL-C and high TG blood levels. As this is the first study exploring miR-binding SNPs in *GALNT2*, future research should examine the function of hsa-miR-1260, which is hypothesized to bind to rs16851339.

Although the results of a previous study demonstrated that *LPL* rs1059611 was associated with HDL-C and TG concentrations in the Chinese Han population [27], we found no relationship between this locus and lipid levels. Moreover, studies reported that rs1059611 influences plasma lipid concentrations and interacts with plasma n-6 PUFA to modulate lipid metabolism [28, 29], indicating the importance of this locus in lipid regulation.

### Combined genetic risk of low HDL-C levels

In this study, pregnant women who carry more risk alleles had a greater risk for low HDL-C levels. Because individual variants can only explain a small part of clinical traits’ heritability, investigation into the combined influence of risk loci may improve genetic risk prediction for diseases [30, 31]. Some studies found evidence that combining multiple common genetic risk variants improves HDL-C predictions in non-pregnant populations [32–34]. In a study of Chinese genetic variants in lipid parameters and dyslipidemia, HDL-C levels decreased from 1.38 mmol/L for individuals carrying three or fewer risk alleles to 1.14 mmol/L for individuals carrying all eight risk alleles (*MLXIPL* rs17145738, *LPL*rs326, *LIPC* rs1800588 and *CETP* rs3764261) [35]. These results support our findings that HDL-C risk alleles have additive effects on lipid levels in pregnant Chinese women. However, there were still inconsistent results, which demonstrate the lack of an association between the combined risk allele score of relative genes for HDL-C (*SORT1, GCKR, LPL, APOA1, CETP, LDLR, APOE*) and the HDL-C concentration [36].

There are some limitations in the present study. When we analyzed the association between HDL-C level and SNPs, we did not correct for some confounding factors, including hormone levels, especially estrogen, progesterone, and insulin, or changes in life-style, such as less physical exercise during pregnancy, which may greatly influence the results. Additionally, only common variants related to HDL-C were examined, however, more SNPs with relatively low frequencies but important function need to be included. Moreover, the limited sample size may not possess sufficient power to find the related SNPs with small effects on HDL-C.

## MATERIALS AND METHODS

### Ethics statement

The study's protocol was approved by the local Ethics Committee of Peking Union Medical College Hospital, and written informed consent was obtained from each participant.

### Subjects

During the period of 2006 to 2011, we recruited a total of 1,884 participants among outpatients attending the Endocrinology Clinics of Peking Union Medical College Hospital, Beijing, China, including 594 women with GDM. Participants who had previously been diagnosed with hyperlipidemia, preeclampsia, hypertension, or severe systemic disease, or had been prescribed medications affecting lipid metabolism were excluded from the study. Levels of serum glucose, insulin, and lipids (including TG, total cholesterol, LDL-C, and HDL-C) were measured in the pregnant women between 24 and 28 weeks of gestation.

### SNP selection

**Figure 2:**
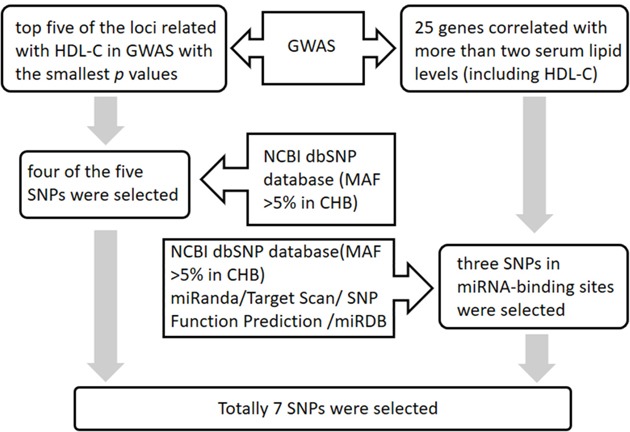
The flowchart of the SNP selection procedure

SNPs were selected according to the following 2 steps: 1) the selection of common HDL-C related loci: We selected genes containing SNPs associated with HDL-C levels (as shown in GWAS). First, the top five of these loci with the smallest *p* values were selected. Then, four (locating at 4 different gene) of the five SNPs with a minor allele frequency (MAF) >5% in the Chinese Han population were identified based on the 1000 genomes browser (http://www.1000genomes.org/), including rs3764261 (*CETP*), rs1532085 (*LIPC*), rs7241918 (*LIPG*) and rs1883025 (*ABCA1*). 2) the selection of miR-binding SNPs: Four genes mentioned above and additional 25 genes that had a statistically significant correlation with more than two serum lipid levels (including HDL-C) were included [37]. A total of 25 genes were analyzed further for the presence of potential miR-binding SNPs. To investigate SNPs in miRNA-binding sites, we used four miRNA target prediction software packages, miRanda (http://www.microrna.org), TargetScan (http://www.targetscan.org/), SNP Function Prediction (https://snpinfo.niehs.nih.gov/), and miRDB (http://mirdb.org/). Only SNPs predicted to have concordant effects on miRNA-binding based on predictions by at least two software packages were chosen. After excluding loci with MAF < 5%, based on the NCBI dbSNP database (http://www.ncbi.nlm.nih.gov/snp/), three SNPs potentially located in miRNA-binding sites of all the 25 candidate genes were included. These loci were in the 3′UTR of nearby genes, *APOC3* (rs4225), *LPL* (rs1059611), and *GALNT2* (rs16851339). Thus a total of 7 SNPs (4 from step 1 and 3 from step 2) were ultimately selected to our study. The selection procedure was shown in Figure 2.

### DNA extraction and genotyping

Total genomic DNA was extracted from whole blood samples obtained from subjects using a Qiagen blood DNA mini kit (QIAGEN, Germany). DNA samples were diluted to a final concentration of 20-50 ng/uL and stored at −20°C. Genotyping was performed using the SequenomMassARRAY (Agena Bioscience, USA). As a genotyping quality-control measure, a second round of analysis was performed on 10% of the samples, which were randomly selected. All replicate assay results were in complete accordance with the original analysis. The genotyping success rates were 96.1%, 95.8%, 96.3%, 96.3%, 97.1%, 96.1% and 97.3% for rs3764261, rs1532085, rs7241918, rs1883025, rs4225, rs1059611 and rs16851339, respectively.

### Statistical analysis

All quantitative variables in this study were non-normally distributed and expressed as median and interquartile range and nonparametric tests were used to analyze differences between groups. Three types of genotype in specific locus were given codes of 0, 1, and 2 (A1A1 vs A1A2 vs A2A2) and a multiple linear regression model was used to investigate the individual effect of these genes on serum lipid levels during pregnancy, after adjusting for age, gestational week, pre-pregnancy BMI and state of GDM or HOMAIR. The regression coefficients (b) with 95% confidence intervals (CIs) are presented. To explore the compound effects of multiple risk alleles on HDL-C, data of which were normal distribution after splitting into three groups, subjects were classified into one of the groups, which were given codes of 1, 2, and 3 (corresponding to 0-2, 3-5, and 6-8 risk alleles, respectively) and comparisons between groups were tested by One - Way ANOVA analysis and LSD test.

A two-tailed *P* value < 0.05 was considered statistically significant. Hardy-Weinberg equilibrium of the genotypes for each SNP was tested using a Chi-square test.

Statistical analyses were performed using SPSS software version 20.0.

## Abbreviations

HDL-C: high-density lipoprotein-cholesterol
GWAS: genome-wide association studies
FPG: fasting plasma glucose
LDL-C: low-density lipoprotein-cholesterol
TG: triglyceride
CHD: coronary heart disease
SNP: single-nucleotide polymorphism
miRNA: microRNA
3′UTR: 3′-untranslated region
miR-binding-SNP: SNP in miRNA-binding site
MAF: minor allele frequency
PCR: polymerase chain reaction
SAP: Shrimp alkaline phosphatase
CI: confidence interval
PAGE: Population Architecture using Genomics and Epidemiology
AMD: age-related macular degeneration
OR: odds ratio
